# “Comparative safety and efficacy of robotic TAPP and IPOM techniques in ventral hernia repair: a systematic review and meta-analysis of Short-term Outcomes”

**DOI:** 10.1007/s10029-025-03454-0

**Published:** 2025-08-19

**Authors:** Ahmed Abdelsamad, Ibrahim Khalil, Khaled Ashraf Mohamed, Aya Sayed Ahmed Said Serour, Mohammed Khaled Mohammed, Noureldin Mostafa, Youssef Osama Badie, Zeyad M. Wesh, Alaeldin Mohamedsami Mohamedosman Ali, Florian Gebauer

**Affiliations:** 1 Department of Surgery II, University of Witten/Herdecke, Witten, Germany; 2 Deputy head of the oncological surgery department, Section head of robotic surgery, Knappschaft Vest-Hospital, 45657 Recklinghausen, Germany; 3 Faculty of Medicine, Alexandria University, Alexandria, 5372066 Egypt; 4 General surgery Department, Cairo University hospitals, Cairo, Egypt; 5 Faculty of Medicine, Cairo University, Cairo, Egypt; 6 Faculty of Medicine, Ibn Sina University, Khartoum, Sudan , Khartoum, Sudan; 7 Head of Oncological Surgery Department, Helios University Hospital, Wuppertal, Germany

**Keywords:** Robotic ventral hernia repair, r-TAPP, r-IPOM/rIPOM+, Perioperative complications, Meta-analysis

## Abstract

**Background:**

Robotic-assisted ventral hernia repair has gained popularity for its enhanced precision and visualization. Two main approaches—r-IPOM and r-TAPP—differ in technique and risk profiles. r-IPOM/+ is technically simpler and preferred for larger defects, but may increase seroma and bowel-related complications. Conversely, r-TAPP employs extraperitoneal mesh positioning, potentially reducing postoperative complications. This meta-analysis aimed to compare perioperative outcomes between r-IPOM and r-TAPP, focusing on seroma, surgical site infection (SSI), and hernia defect closure. Secondary outcomes included recurrence, reoperation, operative time, and hospital stay.

**Methods:**

A systematic meta-analysis, including 11 studies and 1001 patients, was performed. Pooled event rates and mean differences were calculated using random-effects models. Subgroup analyses based on mesh type and meta-regression assessing the impact of defect closure on complication rates were conducted. Evidence certainty was evaluated using the GRADE approach.

**Results:**

Both approaches achieved high defect closure rates (r-IPOM+: 98%, r-TAPP: 99%; *p* = 0.9). Seroma and hematoma rates were low without significant differences; however, r-IPOM showed a slightly higher trend. r-TAPP demonstrated a significantly lower Surgical site infection (SSI) rate (1%) compared to (r-IPOM 4%, *p* = 0.02), although these complications themselves did not differ significantly between groups, indicating a possible but unconfirmed association. Recurrence, reoperation, operative time, and hospital stay were comparable. Meta-regression indicated a non-significant trend toward fewer complications with higher closure rates (*p* = 0.09). The GRADE assessment rated the certainty of evidence as high across all outcomes.

**Conclusion:**

Both r-TAPP and r-IPOM are effective and safe for robotic ventral hernia repair. A significant inverse correlation between closure rates and complication rates underscores the importance of complete, tension-free closure. While perioperative outcomes are largely comparable, r-TAPP may reduce infection risk likely due to lower seroma and hematoma rates. Approach selection should be guided by patient factors, anatomical considerations, and surgical expertise.

**Supplementary Information:**

The online version contains supplementary material available at 10.1007/s10029-025-03454-0.

## Introduction

Robotic-assisted techniques have revolutionized ventral hernia surgery by offering enhanced dexterity, three-dimensional visualization, and ergonomic advantages [1]. Among the most commonly adopted robotic approaches are the intraperitoneal onlay mesh repair (r-IPOM) and the transabdominal preperitoneal repair (r-TAPP) [2]. While both techniques have demonstrated feasibility and safety, their comparative efficacy and short-term outcomes remain a subject of ongoing debate.

The r-IPOM approach, which involves placing the mesh intraperitoneally with (rIPOM+) or without defect closure, has been favored for its technical simplicity and shorter operative time. It is often selected in larger hernia defects, especially when defect closure is not feasible [3]. However, r-IPOM has been associated with higher rates of hematoma and seroma, as well as a greater risk of surgical site infections and bowel adhesions [4]. These complications are largely attributed to the direct contact of the mesh with the intra-abdominal viscera, particularly when barrier-coated meshes are not used [5]. The use of tacker-based fixation, which is common in r-IPOM, may further contribute to increased postoperative pain and discomfort [6, 7]. Despite these concerns, r-IPOM remains a practical option in complex cases requiring rapid mesh deployment and broad coverage [8].

In contrast, the r-TAPP approach involves accessing the preperitoneal space to place the mesh between the peritoneum and abdominal wall, avoiding direct contact with the bowel [9]. This technique allows for better mesh integration and reduced adhesion formation risk due to the prosthetic material’s extraperitoneal placement [10]. Additionally, r-TAPP is often associated with lower rates of hematoma and seroma formation [11]. Pain outcomes also tend to favor r-TAPP, as mesh fixation is typically performed using absorbable or non-absorbable sutures, which are associated with less nerve irritation and chronic pain compared to tacks [12]. However, r-TAPP can be technically more demanding and time-consuming, with operative times often exceeding those of r-IPOM, particularly in patients with prior surgeries or larger hernias where adequate dissection of the preperitoneal space becomes challenging [13].

While both approaches are widely implemented, the evidence base comparing their perioperative outcomes remains heterogeneous, with limited head-to-head comparisons and variable reporting standards across studies [14–24]. Postoperative complications such as seroma, hematoma, and surgical site infection are among the most frequently reported early adverse events in hernia surgery [25]. Additionally, closure of the hernia defect, when feasible, is considered a key factor in reducing tension on the mesh and lowering recurrence rates [26]. Nonetheless, the feasibility of closure may vary significantly between techniques, with r-TAPP offering more controlled anatomical conditions for sutured closure in small- to moderate-sized defects [27], while r-IPOM is often preferred in larger or more complex hernias where closure is not possible [14, 28–30].

In this meta-analysis, we collected data from recent primary studies to compare the short-term clinical outcomes of r-TAPP versus r-IPOM/+ in the treatment of ventral hernias. The primary outcomes include the incidence of seroma, hematoma, SSI, and successful defect closure. Secondary outcomes include recurrence, reoperation, operative time, and length of hospital stay.

## Methods

### Search strategy

This systematic review and meta-analysis was conducted following the Items for Systematic Reviews and Meta-Analyses Protocols (PRISMA) checklist [31] and the principles of Cochrane Handbook for Systematic Reviews of Interventions version 6.2, We used online databases: SCOPUS, Web of Science, Medline (PubMed), and Cochrane, till February 2025. The search strategy was based on Medical Subject Headings (MeSH) and included the following keywords and Boolean operators: “Robotic” AND “IPOM OR “intraperitoneal onlay mesh” OR “intraperitoneal onlay mesh plus” OR “r-IPOM” OR “IPOM+” AND “TAPP OR “Transabdominal preperitoneal repair” OR “preperitoneal” OR “sublay mesh” AND “Ventral hernia” OR “Hernia”, to ensure reaching out to all the potential included studies. A word cloud in Figure S summarizes the most frequent terms from all retrieved titles and abstracts (Table S).

### Study selection

The study was structured according to the PICO framework. P: Ventral hernia patients, I: Robotic mode of repair implementing rIPOM (Intraperitoneal Onlay Mesh repair)/rIPOM+, C: Robotic mode of repair implementing rTAPP (Trans-abdominal preperitoneal mesh repair), O: outcomes included a range of surgical endpoints, such as seroma, operative time, intraoperative bleeding, recurrence, SSI, closure of the hernia defect, and excision of the hernia sac, encompassing both short-term and long-term clinical results.

This comprehensive literature search yielded a total of 58 studies that were screened for eligibility. After duplicate removal using EndNote, 28 studies remained and were screened based on titles and abstracts. Full-text assessment was then performed to identify primary studies reporting outcomes of either r-TAPP, r-IPOM, or both in the context of ventral hernia repair.

Studies were excluded if they met any of the following criteria: (1) conducted on animal models, (2) focused on hernia types other than ventral hernia, (3) employed surgical techniques other than r-IPOM or r-TAPP, or (4) lacked sufficient outcome data for analysis.

**Fig. 1:**
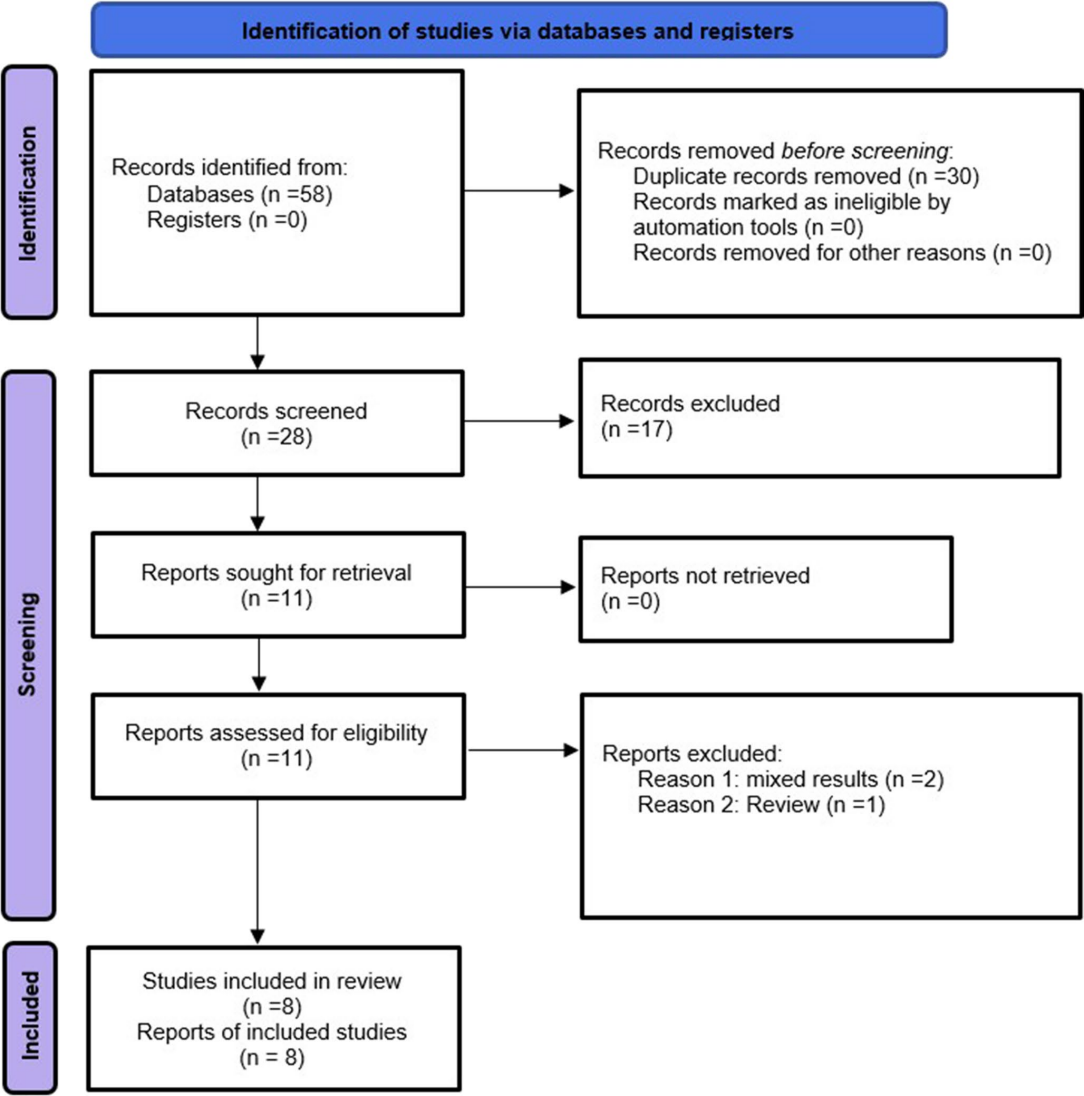
PRISMA 2020 flow diagram for new systematic reviews which included searches of database and registers only

Ultimately, eleven studies fulfilled all eligibility criteria and were included in the meta-analysis: Gockal et al. 2019 [14], Kudsi et al. 2020 [15], Wijerathne et al. [16] Bindal et al. 2024 [17], Kennedy et al. 2018 [18], Chelliah et al. 2024 [19], Ferraro et al. 2023 [20], Baur et al. 2021 [21], Bauer et al. 2024 [22], Kudsi et al. [23], and Gokcal et al. 2020 [24]. The study selection process is illustrated in the PRISMA flow diagram, as shown in Fig. 1 [31].

### Selection and screening

Titles, abstracts, AND full-texts were screened by Y.O.B, A.M.A, and N.M.A, and further reviewed by A.S.A and Z.M.W, to ensure meticulous study selection according to the criteria mentioned. They also extracted the data using Excel sheets to obtain outcomes and baseline characteristics.

### Quality assessment and risk of bias

A.M.A and N.E.M assessed the quality of included studies using the Newcastle-Ottawa Scale (NOS) used for the assessment of case-control studies and cohort studies [32]. NOS assesses each study by dividing it into 3 elements: selection, comparison, and exposure or outcome, whether the study is a case-control or cohort study, respectively. For each category, each fulfilled item scores one star, and for comparability, the maximum score is two stars. Study quality is determined by the number of stars. Studies of high-quality score (7–9), moderate quality studies score (4–6), whereas low-quality studies score (0–3), The assessment of nine studies was conducted revealing that, One study was rated as high-quality and eight of the studies were rated as moderate quality as shown in Table 1. For assessment of the risk of bias, we used the ROBINS-I V2 tool to evaluate our studies since most of them were non-randomized cohort studies. Each study was assessed across seven domains: confounding, selection of participants, classification of interventions, deviations from intended interventions, missing data, measurement of outcomes, and selection of the reported result. Risk levels are color-coded as low, moderate, serious, or critical. Most studies were rated as having a low to moderate risk of bias, with no studies judged to be at critical risk. The overall assessment of risk of bias is considered to be low.

**Table 1:** Quality assessment of the included studies according to the Newcastle–Ottawa Scale (NOS) for case–control and cohort studies

Study ID	Selection				Comparability		Exposure			AHRQ standards
	Representativeness of the exposed cohort	Selection of the non-exposed cohort	Ascertainment of exposure	Demonstration that outcome of interest was not present at start of study	Study controls for age	Study controls for any additional factor	Assessment of outcome	Was follow-up long enough for outcomes to occur	Adequacy of follow up of cohorts	
Bindal 2024	*		*	*				*	*	
Kudsi c 2020	*		*	*			*	*	*	*
Gockal 2019	*		*	*				*	*	
Kennedy 2018	*		*	*					*	
Chelliah 2024	*		*	*					*	
Ferraro 2023	*		*	*					*	
Baur 2021	*		*	*					*	
Baur 2024	*		*	*					*	
Kudsi R 2021	*		*	*			*	*		

We used the AMSTAR 2 tool to appraise the methodological quality of our Meta-analysis [33]. Two reviewers independently evaluated the study across 16 domains. Any discrepancies were first discussed between the reviewers, and if no consensus was reached, a third reviewer (AA) was consulted to resolve the disagreement. Overall confidence ratings were determined according to AMSTAR 2 guidelines [34], as per Table 2.

**Table 2:** This table is designed to facilitate the structured evaluation of systematic review and meta-analysis (SRMA), including non-randomized studies, based on the AMSTAR 2 (A measurement tool to assess systematic reviews, version 2) instrument

No.	AMSTAR 2 Item	Rating (Yes/Partial/No)	Comments
1	Did the research questions and inclusion criteria include the components of PICO?	★	
2	Did the review have an explicit statement that the methods were established prior to the conduct of the review (e.g., a protocol)?	★	
3	Did the review authors use a comprehensive literature search strategy?	★	MeSH database was used.
4	Did the review authors perform study selection in duplicate?	★	
5	Did the review authors perform data extraction in duplicate?	★	
6	Did the review authors provide a list of excluded studies and justify the exclusions?	★	
7	Did the review authors describe the included studies in adequate detail?		
8	Did the review authors use a satisfactory technique for assessing the risk of bias (RoB) in individual studies?	★	
9	Did the review authors report on the sources of funding for the studies included in the review?		
10	If meta-analysis was performed, did the review authors use appropriate methods for statistical combination of results?	★	
11	If meta-analysis was performed, did the review authors assess the potential impact of RoB in individual studies on the results?	★	
12	Did the review authors account for RoB in individual studies when interpreting/discussing the results of the review?	★	
13	Did the review authors provide a satisfactory explanation for, and discussion of, any heterogeneity observed in the results?	★	
14	If they performed quantitative synthesis, did the review authors carry out an adequate investigation of publication bias?		
15	Did the review authors report any potential sources of conflict of interest, including any funding they received for conducting the review?	★	
16	Were the review authors’ conclusions supported by the results and did they consider the limitations of the included studies?	★	

### Study endpoints

Primary outcomes of interest were postoperative seroma formation, hematoma incidence, SSI, and successful hernia defect closure. These were selected based on their clinical relevance in early postoperative recovery and potential implications for long-term outcomes. Secondary outcomes included operative time, hospital stay, bowel-related complications, recurrence, and reoperation.

### Statistical analysis

We conducted meta-analyses for both binary and continuous outcomes using random-effects models to account for both within-study and between-study heterogeneity. All analyses were performed using the DerSimonian-Laird method for estimating between-study variance (tau²) as implemented in the ‘meta’ and ‘metafor’ packages in R version 4.0.0 [35, 36].

For binary outcomes (hematoma, seroma, surgical site infection, recurrence, and hernia defect closure), proportions were used as the summary measure with 95% confidence intervals calculated using the Clopper-Pearson exact method, which provides more reliable estimates for rare events [37]. Studies reporting zero events were incorporated by applying a continuity correction of 0.5 to both the numerator and denominator [38]. Between-group comparison was performed using the test for subgroup differences, with chi-squared statistics and corresponding p-values to determine whether differences between r-IPOM and r-TAPP approaches were statistically significant [39].

For continuous outcomes (length of hospital stay, operative time, and consule time), the mean difference (MD) was used as the effect measure with 95% confidence intervals. Weighted means were calculated for each surgical approach by weighting individual study means by their respective sample sizes. The variance of the mean difference was computed as the sum of the variances of each group’s mean [40].

Heterogeneity across studies was quantified using I² statistics for both binary and continuous outcomes, with values of 25%, 50%, and 75% considered to represent low, moderate, and high heterogeneity, respectively [41]. To further explore potential sources of heterogeneity for key outcomes, meta-regression analyses were performed. For instance, the influence of defect closure rate (%) as a continuous moderator variable on the overall complication rate (%) was examined using random-effects meta-regression models implemented in the ‘metafor’ package. The statistical significance of moderator variables was determined by the p-value of their respective regression coefficients. The statistical significance of the overall effect was assessed using Z-tests, with *p* < 0.05 considered statistically significant.

Comprehensive sensitivity analyses were conducted, including leave-one-out analyses to identify influential studies and alternative methods for heterogeneity estimation (maximum likelihood and restricted maximum likelihood) [42]. Publication bias was assessed using contour-enhanced funnel plots. All results were visualized using forest plots showing individual and pooled effect estimates with corresponding confidence intervals.

#### Assessment of evidence quality

We evaluated the certainty of evidence for each outcome using the GRADE (Grading of Recommendations Assessment, Development, and Evaluation) approach. The certainty was categorized as high, moderate, low, or very low based on risk of bias, inconsistency, indirectness, imprecision, and other considerations [43].

## Results

A total of 11 primary studies, encompassing 1001 patients, were included in this meta-analysis. All outcomes were analyzed when data were available for comparative evaluation between r-IPOM and r-TAPP approaches. The results are presented in structured sections, according to clinical categories, including all variables, mesh type subgroup analysis, meta-regression, and sensitivity analyses.

Table 3 summarizes the characteristics of the included studies and patient populations. It provides details on study year, country, type of robotic repair (r-IPOM or r-TAPP), number of patients, demographic data (age, sex, BMI), comorbidities (hypertension, coronary artery disease, COPD, diabetes, smoking), and type of hernia. Notably, the distribution of hernia types and mesh materials varied across studies, and complete baseline data were unavailable for all study variables.

**Table 3:** Patients’ characteristics

Study ID	Year	Country	Type of Repair	Number of Patients	Age (year)	Sex (F/M)	BMI	Comorbidities					Type of hernia	Adhesions	Pain	Bowel Obstruction	Recurrence	Duration of follow up/Months	Time of Hospital Stay/Days
								Hypertension	coronary artery disease	COPD	Diabetes Mellitus	Smoking			According to VAS score or number of patients complained				
Bindal	2024	India	r-IPOM	-	-	-	-	-	-	-	-	-	-	-	-	-	-	-	-
			r-TAPP	33	42.91	21/12	30.48	6	3	6	6	6	ventral	0	5.77	N/A	N/A	-	1.18
Kudsi R	2020	USA	r-IPOM	5	58.2	3/2	34.9	3	0	1	2	3	lateral incisional	N/A	0	N/A	0	33.3 ± 18.618	-
			r-TAPP	8	57	5/3	28.9	4	1	4	2	3	lateral incisional	N/A	0	N/A	0	33.3 ± 18.618	-
Gockal	2024	USA	r-IPOM	104	50.9	44/60	31	45	45	52	22	52	ventral	N/A	21	3	0	3	0
			r-TAPP	104	50.2	41/63	31.03	61	61	44	22	44	ventral	N/A	16	0	0	3	0
Kennedy	2018	USA	r-IPOM	27	45.6	22/5	31.8	46	6.8	2.6	21.1	13.4	ventral	N/A	N/A	N/A	N/A	1	-
			r-TAPP	36	44.3	24/12	31	30.9	5.3	0	23.4	19.8	ventral	N/A	N/A	N/A	N/A	1	-
Chelliah	2024	USA	r-IPOM	63	60	32/31	32	-	-	-	-	-	ventral and incisional	N/A	N/A	N/A	0	13.5	0
			r-TAPP	199	56	48/151	31	-	-	-	-	-	ventral and incisional	N/A	N/A	N/A	0	14.9	0
Ferraro	2023	Italy	r-IPOM	7	-	-	-	-	-	-	-	-	ventral and incisional	N/A	N/A	-	0	1	2.4
			r-TAPP	32	-	-	-	-	-	-	-	-	ventral and incisional	N/A	N/A	0	0	1	2.4
Baur	2021	Switzerland	r-IPOM	-	-	-	-	-	-	-	-	-	lateral incisional, umbilical, epigastric and Spieghel	-	-	N/A	-	1.5	-
			r-TAPP	88	52.3	25/63	30.7	43	10	8	13	37	lateral incisional, umbilical, epigastric and Spieghel	N/A	2.3	N/A	0	1.5	1.5
Bauer	2024	Germany	r-IPOM	1	-	-	-	-	-	-	-	-	incisional hernia.lateral hernia,	N/A	N/A	N/A	N/A	1-1.5	-
			r-TAPP	18	71	11/9	27.5	-	-	-	-	-	incisional hernia.lateral hernia,	N/A	N/A	N/A	N/A	1-1.5	3.39
Kudsi.C	2020	USA	r-IPOM	90	47.9±14.5	48/42	31.3±6.3	35	6	9	11	17		N/A	N/A	N/A	0	3	-
			r-TAPP	108	50.2±14.2	30/78	30.5±5.7	47	3	9	15	26	Midline hernia	N/A	N/A	N/A	0	3	-

The mesh characteristics used across included studies showed that hernial defect closure was consistently performed in both r-IPOM + and r-TAPP groups, primarily using barbed sutures such as V-Loc™ or Stratafix™. Macroporous polypropylene meshes were most commonly employed, with occasional use of PVDF, monofilament, or composite materials. Fixation methods varied by technique and mesh type, including sutures, tackers, glue, or self-fixating meshes. Despite these differences, mesh type and fixation strategy were generally consistent within each study, supporting comparability between surgical approaches, as demonstrated in Table 4.

**Table 4:** Mesh characteristics

Study ID	Year	Country	Type of Repair	Defect closure	Defect closure material	Type of Mesh Used	Method of Mesh Fixation
Bindal	2024	India	r-TAPP	Yes	V-Loc^®^	macroporous polypropylene	non-absorbable sutures
Kudsi R	2020	USA	r-IPOM	Yes (3) No (2)	(Stratafix 0TM on CT-1 needle, Ethicon, Somerville, NJ, USA)	N/A	sutures (95) tackers (2) Both (7)
			r-TAPP	Yes	N/A	N/A	Sutures
Gockal	2024	USA	r-IPOM	Yes	(Stratafx 0TM on CT-1 needle, Ethicon, Somerville, NJ, USA)	Macroporous (Symbotex™ 92.3%, Synecor™% 3.8) Microporous (Proceed™ 1.9%)	Interrupted sutures 6
			r-TAPP	Yes	(Stratafx 0TM on CT-1 needle, Ethicon, Somerville, NJ, USA)	Macroporous (Symbotex™ 38.8, ProGrip™ 58.7%, Phasix™ 1%, Synecor™ 1%, Synecor Pre™ 1%)	Running sutures (V-Loc) or absorbable tackers (AbsorbaTack™)
Kennedy	2018	USA	r-IPOM	Yes	V-Loc sutures	Macroporous Polypropilene	V-Loc sutures
			r-TAPP	Yes	(Stratafx 0TM on CT-1 needle, Ethicon, Somerville, NJ, USA)	Macroporous Polypropilene	V-Loc sutures
Chelliah	2024	USA	r-IPOM	Yes	long-term absorbable suture	monoflament, macroporous	absorbable suture
			r-TAPP	Yes	long-term absorbable suture	monoflament, macroporous	absorbable suture
Ferraro	2023	Italy	r-IPOM	Yes	V-LocTM,	Coated	3–0 V-Loc
			r-TAPP	Yes	V-LocTM	Macroporous Polypropilene (6) Coated (27)	sutures or synthetic glue
Baur	2021	Switzerland	r-IPOM	-	N/A	-	sutures or synthetic glue
			r-TAPP	Yes (75) No (13)	None 13 sutures 75	Macroporous (DynameshEndolapVisible 73,Progrip 15)	None 1 Vicryl suture (82) V-Loc suture (5)
Bauer	2024	Germany	r-TAPP	Yes	N/A	PVDF	N/A
Kudsi.C	2020	USA	r-IPOM	Yes (79) No (11)	Stratafx 0TM on CT-1 needle, Ethicon, Somerville, NJ, USA	Macroporous (Polypropilene 2, Polyester 86) microporous (ePTFE 2)	Sutures
			r-TAPP	Yes (104) No (4)	N/A	Macroporous (Polypropilene 11,polyester 95) Microporous (ePTFE 2)	sutures (82) self-gripping (24) None (2)

### Hernial defect closure rates

**Fig. 2:**
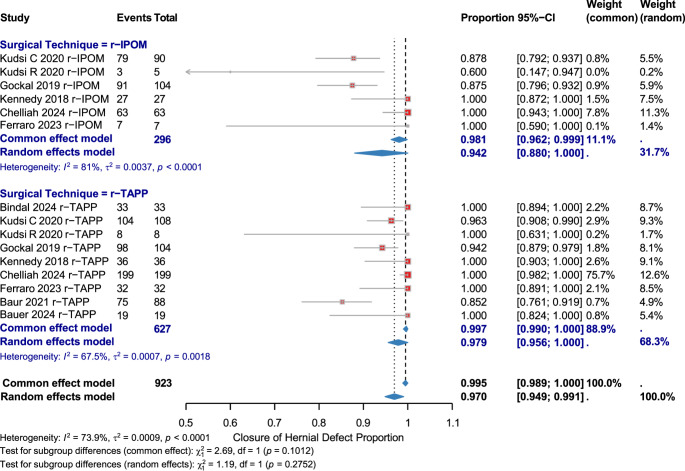
Closure of hernial defect by surgical technique

Fifteen studies (923 patients) from eight publications reported on hernial defect closure rates for r-IPOM+ (6 studies, 296 patients) and r-TAPP (9 studies, 627 patients). High heterogeneity (I² = 73.9%) warranted the use of the random-effects model. The pooled closure rate was 0.942 [95% CI: 0.880–1.000] for r-IPOM (I² = 81%), and 0.979 [95% CI: 0.956–1.000] for r-TAPP (I² = 67.5%). The combined pooled rate was 0.970 [95% CI: 0.949–0.991]. No significant subgroup difference was found between techniques (χ² = 1.19, df = 1, *p* = 0.2752). (Fig. 2, Supplementary Figs. 1)

### Complication rates

#### Hematoma

**Fig. 3:**
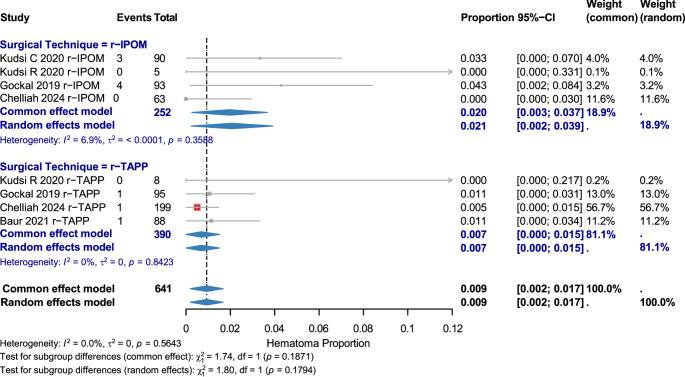
Hematoma complications by surgical technique

Eight studies (641 patients) showed low heterogeneity (I² = 0.0%). Overall rate: 0.009 [95% CI: 0.002–0.017]. r-IPOM had a rate of 0.020 [95% CI: 0.003–0.037]; r-TAPP: 0.007 [95% CI: 0.000–0.015]. No significant subgroup difference (χ² = 1.80, *p* = 0.1794). (Fig. 3)

#### Long-term recurrence

**Fig. 4:**
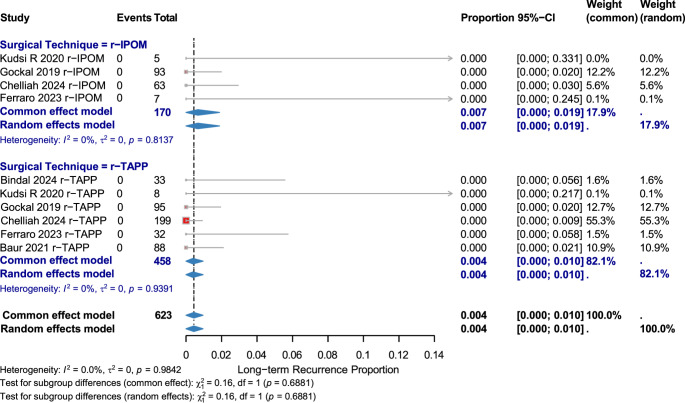
Long−term recurrence by surgical technique figure 5. Reoperation Rate by Surgical Technique

Ten studies (623 patients) reported a low recurrence rate: 0.004 [95% CI: 0.000–0.010], with no heterogeneity. r-IPOM: 0.007 [95% CI: 0.000–0.019]; r-TAPP: 0.004 [95% CI: 0.000–0.010]. No subgroup difference (χ² = 0.16, *p* = 0.6881). (Fig. 4)

#### Reoperation

**Fig. 5:**
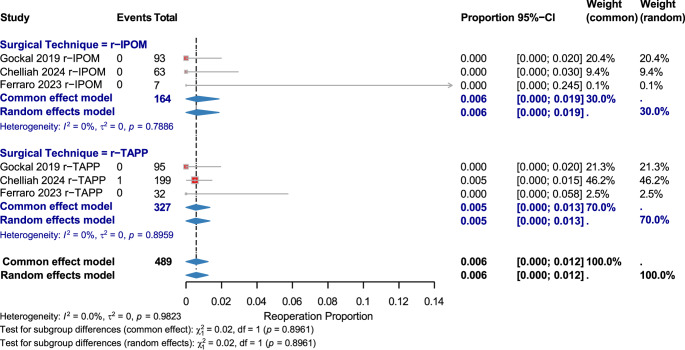
Reoperation Rate by Surgical Technique

Six studies (489 patients) reported an overall rate of 0.006 [95% CI: 0.000–0.012] with no heterogeneity. r-IPOM: 0.006 [95% CI: 0.000–0.019]; r-TAPP: 0.005 [95% CI: 0.000–0.013]. No subgroup difference (χ² = 0.02, *p* = 0.8961). (Fig. 5)

#### Seroma

**Fig. 6:**
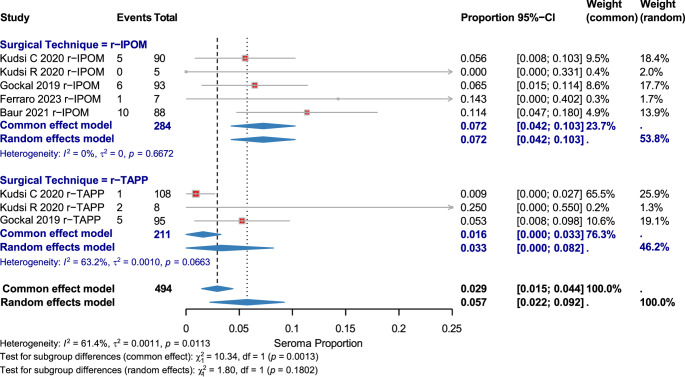
Seroma Complications by Surgical Technique

Eight studies (494 patients) reported moderate heterogeneity (I² = 61.4%). Overall seroma rate: 0.057 [95% CI: 0.022–0.092]. r-IPOM: 0.072 [95% CI: 0.042–0.103]; r-TAPP: 0.033 [95% CI: 0.000–0.082]. No significant subgroup difference (χ² = 1.80, *p* = 0.1802). (Fig. 6, Supplementary Figs. 2)

#### Surgical Site Infection (SSI)

**Fig. 7:**
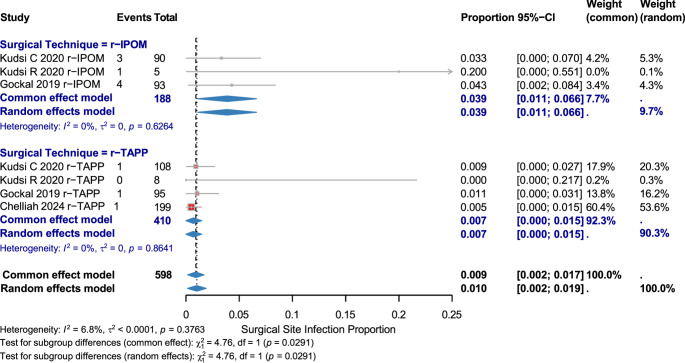
Surgical Site Infection by Surgical Technique

Seven studies (598 patients) showed a low overall SSI rate: 0.009 [95% CI: 0.002–0.017]. r-IPOM: 0.039 [95% CI: 0.011–0.066]; r-TAPP: 0.007 [95% CI: 0.000–0.015]. The subgroup analysis revealed a statistically significant difference favoring r-TAPP (χ² = 4.76, *p* = 0.029). (Fig. 7)

### Other parameters

#### Console time

**Fig. 8:**
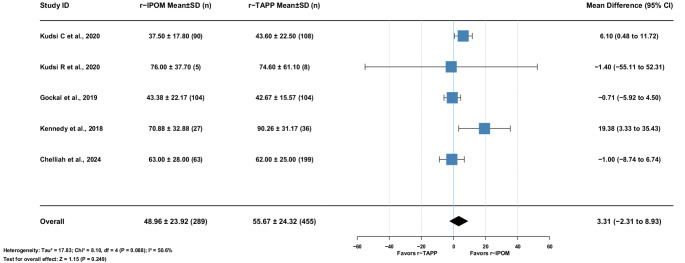
Mean Difference r−TAPP vs. r−IPOM for Console Times (minutes)

Five studies (744 patients) showed moderate heterogeneity (I² = 50.6%). The mean difference was 3.31 min [95% CI: −2.31–8.93], not statistically significant (*p* = 0.249). (Fig. 8)

#### Length of hospital stay

**Fig. 9:**
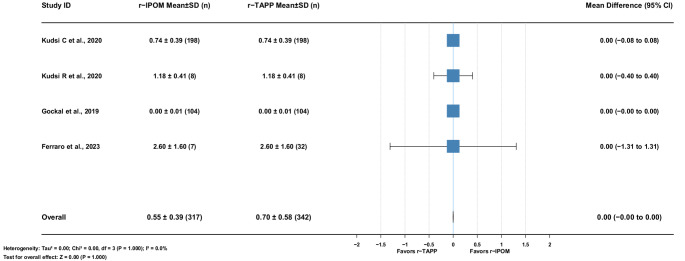
Mean Difference r−TAPP vs. r−IPOM for Length of Hospital Stay (days)

Four studies (659 patients) reported no heterogeneity. Mean difference: 0.00 days [95% CI: −0.00–0.00], *p* = 1.000. (Fig. 9)

#### Operative time

**Fig. 10:**
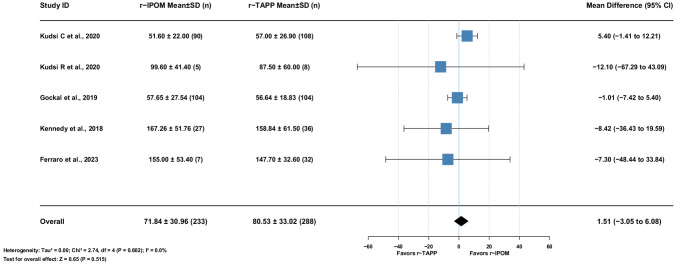
Mean Difference r−TAPP vs. r−IPOM for Operative Times (minutes)

Five studies (521 patients) showed no heterogeneity. Mean difference: 1.51 min [95% CI: −3.05–6.08], *p* = 0.515. (Fig. 10)

### Complication Rates by Mesh Type – Subgroup Analysis

Subgroup analyses comparing non-coated (mostly macroporous in our Meta-analysis) meshes with coated mesh types did not yield any statistically significant differences across the evaluated complications, including recurrence, hematoma, seroma, surgical site infection (SSI), or reoperation rates. Due to inconsistent definitions and considerable heterogeneity in mesh classification, along with the absence of meaningful statistical differences, the results of this subgroup analysis have been omitted from the main results and supplementary materials, as per Supplementary Fig. 3.

### Meta-regression

**Fig. 11:**
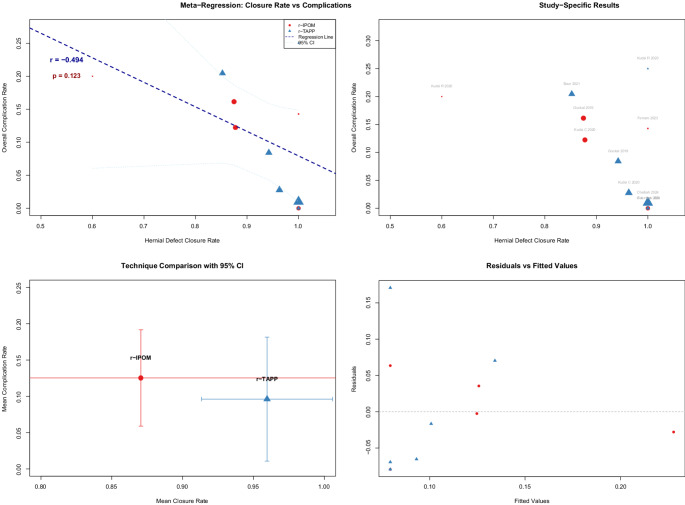
Meta−Regression: Closure Rate vs. Complications

Meta-regression investiNgated the relationship between closure and complication rates. The unweighted model did not reach significance (*r* = −0.494, *p* = 0.1226), as shown in (Fig. 11, Supp. Figure 4). However, a sample-size weighted model showed a significant inverse correlation:

*Complication Rate = 1.0290–1.0121 × Closure Rate* (R² = 0.747, *p* = 0.0006), suggesting that higher closure rates predict lower complication rates.

### Sensitivity analyses

Leave-one-out analyses demonstrated robustness for most outcomes. In seroma analysis, excluding Kudsi C 2020 (r-TAPP) or Baur 2021 (r-IPOM) reduced heterogeneity notably. For SSI, omitting Kudsi C 2020 or Gockal 2019 (both r-IPOM) eliminated heterogeneity. Console time heterogeneity decreased from 50.6 to 18.1% after removing Kennedy et al. 2018. Other outcomes (hematoma, recurrence, reoperation, hospital stay, operative time) were not notably affected by single-study exclusion.

### Publication bias assessment

**Fig. 12:**
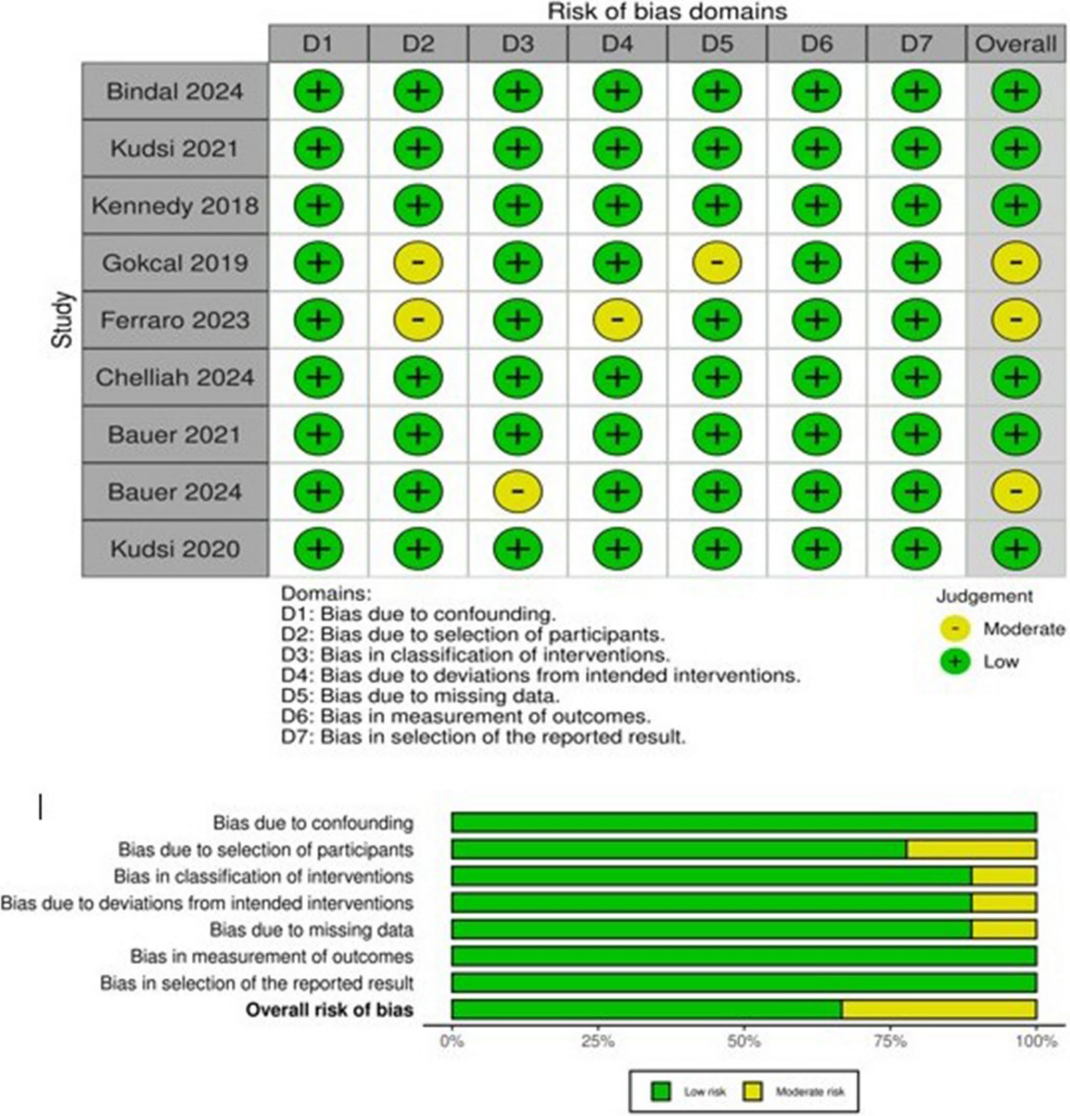
ROBINS-I Risk of Bias Assessment. Summary of bias domains across included non-randomized studies using the ROBINS-I tool. Green indicates low risk; yellow indicates moderate risk. The bar chart below shows the proportion of risk levels across all domains.

The risk of bias for non-randomized studies was evaluated using the ROBINS-I tool, covering seven domains. As illustrated in Fig. 12, most studies showed a low to moderate risk of bias. No study was assessed to have a serious or critical risk across any domain. The most frequent sources of moderate risk were related to participant selection and intervention classification, underscoring the inherent limitations of observational data despite overall acceptable quality.

### Methodological quality assessment

Using the AMSTAR 2 tool, the methodological quality of our meta-analysis was evaluated across 16 domains. The overall score achieved was 13 out of 16, corresponding to 81%, which reflects a high methodological quality. According to established AMSTAR 2 interpretation criteria, a score exceeding 70% is considered indicative of robust methodological rigor. This result supports the reliability of our systematic approach and strengthens the validity of the findings presented in this meta-analysis.

### GRADE assessment of evidence quality

The quality of evidence for all primary and secondary outcomes was assessed using the GRADE approach. Overall, the certainty of evidence was rated as high for most clinical outcomes, including hernia defect closure rates, hematoma formation, recurrence, reoperation, seroma formation, surgical site infection, and operative time across both r-IPOM and r-TAPP groups. Console time and hospital stay outcomes were graded as high certainty, despite moderate inconsistency (I² >50%) noted across some analyses. No outcomes were downgraded due to serious risk of bias, indirectness, or imprecision. These results suggest that the findings of this meta-analysis are supported by robust and reliable evidence, as shown in Table 5.

**Table 5:** GRADE assessment

Outcome	No. of studies	Study Design	Risk of Bias	Inconsistency	Indirectness	Imprecision	Other Considerations	Number of Patients	Effect (95% CI)	Certainty	Importance
HERNIAL DEFECT CLOSURE RATES (r-IPOM)	6	Observational	low	I² = 66.5%	Non	Non	Non	284	0.97, 1.00	high	high
HERNIAL DEFECT CLOSURE RATES(r-TAPP)	8	Observational	Low	I² = 66.5%	Non	Non	Non	518	0.98, 1.00	high	high
Hematoma (r-IPOM)	2	Observational	Low	I² = 0.0%	Non	Non	Non	109	0.00, 0.05	high	high
Hematoma (r-TAPP)	3	Observational	Low	I² = 0.0%	Non	Non	Non	457	0.00, 0.01	high	high
Recurrence (r-IPOM)	5	Observational	Low	I² = 0.0%	Non	Non	Non	248	0.00, 0.02	high	high
Recurrence (r-TAPP)	6	Observational	Low	I² = 0.0%	Non	Non	Non	463	0.00, 0.01	high	high
Reoperation (r-IPOM)	3	Observational	Low	I² = 0.0%	Non	Non	Non	174	0.00, 0.01	high	high
Reoperation (r-TAPP)	4	Observational	Low	I² = 0.0%	Non	Non	Non	351	0.00, 0.01	high	high
Seroma (r-IPOM)	4	Observational	Low	I² = 72.8%,	Non	Non	Non	359	0.00, 0.11	high	high
Seroma (r-TAPP)	3	Observational	Low	I² = 18.9%,	Non	Non	Non	130	0.00, 0.05	high	high
Surgical Site Infection (r-IPOM)	3	Observational	Low	I² = 17.4%,	Non	Non	Non	188	0.00; 0.02	high	high
Surgical Site Infection (r-TAPP)	3	Observational	Low	I² = 17.4%,	Non	Non	Non	402	0.00, 0.02	high	high
Console Time (r-IPOM)	5	Observational	Moderate	I² = 50	Non	Non	Non	455	−2.31, 8.93	high	high
Console Time (r-TAPP)	5	Observational	Moderate	I² = 50.6%	Non	Non	Non	289	−2.31, 8.93	high	high
Length of Hospital Stay (r-IPOM)	3	Observational	Moderate	I² = 98.0%	Non	Non	Non	283	−1.09, 0.42	high	high
Length of Hospital Stay (r-TAPP)	3	Observational	Moderate	I² = 98.0%	Non	Non	Non	263	−1.09, 0.42	high	high
Operative Time (r-IPOM)	7	Observational	Low	I² = 83.3%	Non	Non	Non	374	−5.60, 11.49	high	high
Operative Time (r-TAPP)	7	Observational	Low	I² = 83.3%	Non	Non	Non	520	−5.60, 11.49	high	high

## Discussion

This systematic review and meta-analysis aimed mainly to compare perioperative outcomes between robotic-assisted intraperitoneal onlay mesh (r-IPOM) and robotic-assisted transabdominal preperitoneal (r-TAPP) approaches in ventral hernia repair, focusing particularly on seroma formation, surgical site infection (SSI), and hernia defect closure success, while assessing secondary outcomes such as recurrence, reoperation, operative time, and hospital stay.

Clinically, it is well known that tacker use in r-IPOM/+ can lead to more postoperative discomfort compared to suture fixation [7]. While we share this perspective, our synthesis remains limited by the available data.

A potential explanation for the statistically significant higher SSI rate observed in the r-IPOM group may involve the co-occurrence of seromas or hematomas, which have been hypothesized to promote bacterial colonization. This was noted in studies by Kudsi et al. and Gokal et al. However, in our meta-analysis, while the difference in SSI rates reached statistical significance, seroma and hematoma rates did not. These associations should therefore be viewed as hypothesis-generating and require further investigation in prospective studies.

Our findings revealed that both r-IPOM/+ and r-TAPP achieved excellent defect closure rates up to 99% in our included studies, with no significant difference between the two techniques. This outcome underscores the key advantage of robotic assistance in ventral hernia repair, where enhanced instrument articulation, improved ergonomics, and superior visualization collectively facilitate meticulous hernia defect closure [44]. These results align with prior observations by Morrell et al. [45] and Tonelli et al. [46], who similarly documented a high closure rates result in low recurrence rates in robotic hernia repairs. This suggests that robotic technology, regardless of the surgical route, significantly enhances the precision and tensile strength of closure.

Our analysis showed low overall rates of hematoma and seroma for both r-IPOM and r-TAPP, with no statistically significant differences. Although rates were slightly higher in the r-IPOM group (6% seroma, 3% hematoma) versus r-TAPP (1% for both), these differences were not significant, aligning with findings by Kudsi et al. [15, 23]. The robotic platform’s precision and improved hemostasis likely contribute to these favorable outcomes. Nevertheless, the higher hematoma rate in r-IPOM may predispose to infectious complications [20], given the potential of hematomas to promote bacterial growth and impair healing.

In terms of recurrence, our analysis demonstrated no significant difference between r-IPOM and r-TAPP, with observed rates ranging between 0% and 1%. This finding aligns with previous reports, including those by Dixit et al. 2023 [47], who emphasized that recurrence rates in robotic hernia repair are more closely associated with mesh positioning and the quality of defect closure rather than the specific surgical approach utilized. The similarly high defect closure rates achieved in both groups likely contributed to the low recurrence observed across studies. Nevertheless, it is important to acknowledge that the relatively short follow-up periods reported in many of the included studies (ranging from 1 to 33 months) may have limited the ability to capture late recurrences that could emerge over longer-term surveillance.

Reoperation rates were also comparable between r-IPOM/+ and r-TAPP in our analysis, indicating that both techniques are associated with low rates of early postoperative complications requiring surgical reintervention. These results are in line with the findings of Reinhorn et al. [48], who performed a large propensity score-matched analysis using data from the Abdominal Core Health Quality Collaborative registry. Their study evaluated 10,409 patients who underwent unilateral inguinal hernia repair (IHR) between 2012 and 2021. They reported similarly low rates of recurrence and reoperation at one year in the robotic group.

In cases involving massive adhesions, particularly in patients with prior surgeries, extensive adhesiolysis is often required, which increases the risk of inadvertent bowel wall injury. Even when such injuries are recognized intraoperatively and repaired, the risk of bacterial contamination of the surgical field remains substantial [49]. Literature has shown that despite meticulous repair, the probability of subsequent mesh colonization or infection after bowel injury remains high, as bacterial translocation can occur through repaired tissues or via contamination during the procedure itself [50]. Therefore, in patients with extensive adhesions, a history of multiple abdominal surgeries, or those with elevated infectious risk — such as obese individuals, diabetics, or immunocompromised patients (e.g., under chemotherapy or post-transplantation) — the strategy of compartmental separation and placement of the mesh in a preperitoneal or sublay layer, as performed in r-TAPP, may offer significant advantages.

Console time, operative time, and hospital stay were statistically comparable between r-IPOM and r-TAPP. While Kudsi et al. [15] previously noted longer times for r-TAPP due to its complexity, our findings suggest that surgical experience and standardized protocols have minimized these differences. Hospital stay appears more dependent on patient factors and recovery protocols than on the surgical approach, aligning with Yeong et al. (2025) [51], who found perioperative care—not technique—primarily influenced hospitalization duration.

Our meta-regression analysis revealed a statistically significant inverse correlation between hernial defect closure rates and overall complication rates in the sample-size weighted model (R² = 0.747, *p* = 0.0006), indicating that higher closure rates are strongly associated with reduced postoperative complications (Fig. 6, Supplementary Fig. 11). Petersen et al. (2022) [52] similarly found that defect closure during laparoscopic hernia repair reduced recurrence and seroma risk without increasing pain. Christoffersen et al. (2020) [53] also reported significantly lower seroma rates and long-term recurrence with defect closure in a randomized trial. These findings highlight the clinical value of defect closure, warranting further validation through large-scale trials.

In our review, various mesh types were used across studies, including polypropylene, composite, and e-PTFE meshes. However, the heterogeneity in mesh classification and inconsistent reporting of their structural properties (e.g., porosity, coating, or resorption characteristics) limit the interpretability of mesh-related outcomes. To address this, we refrained from performing artificial subgroup analyses based on oversimplified or inconsistent definitions. Moving forward, standardized classification and consistent reporting of mesh properties in robotic hernia surgery studies are essential to enable reliable comparison and more meaningful evidence synthesis.

While Totten et al. [54] found no significant differences in recurrence or infection rates between polypropylene and polyester meshes in a meta-analysis of over 10,000 patients, Saha et al. [55] highlighted that characteristics such as porosity and material influence integration and infection risk, favoring macroporous designs. These contrasting results emphasize the importance of individualized mesh selection based on anatomical and patient-specific factors rather than mesh material alone [56, 57].

An important observation emerging from our analysis is the lack of standardization across robotic hernia repair techniques. Considerable heterogeneity was noted in surgical approaches, mesh types, fixation methods, and defect closure strategies. For instance, r-TAPP repairs most commonly employed barbed sutures such as V-Loc™ for mesh fixation, while r-IPOM repairs often utilized absorbable tacks. In some studies, self-fixating meshes like ProGrip™ were used, whereas others did not report fixation details at all. This variation underscores the need for standardized protocols and consensus guidelines to optimize outcomes and improve comparability across future studies.

### Strengths of our study

Our meta-analysis is among the first to systematically synthesize a comparison between r-IPOM/rIPOM + and r-TAPP in hernia repair using a comprehensive approach, integrating subgroup and meta-regression analyses. By applying rigorous sensitivity analyses, we ensured the robustness of our conclusions, addressing potential sources of heterogeneity with appropriate statistical adjustments.

### Limitations

Several limitations must be acknowledged. Firstly, high heterogeneity was evident in some outcomes, especially operative time and hospital stay, reflecting variations in surgical technique, patient populations, and institutional practices, including mesh types. Although random-effects models and subgroup analyses were employed, this statistical heterogeneity cannot be fully eliminated. Secondly, the reliance on predominantly retrospective studies introduces an inherent risk of bias, and the short follow-up periods limit assessment of long-term outcomes such as chronic pain and late recurrence. Additionally, the lack of uniform surgical definitions and protocols across studies poses another challenge to direct comparisons.

Moreover, although postoperative pain and adhesion formation are highly relevant clinical outcomes, especially when comparing r-IPOM/+ and r-TAPP approaches, these endpoints could not be reliably assessed in our meta-analysis due to inconsistent and incomplete reporting across the included studies. Pain was only explicitly measured in one study (Bindal et al. 2024) using VAS scores, where the r-IPOM/+ group reported significantly more pain. Two additional studies (Gockal et al., 2024; Baur et al., 2021) referenced postoperative pain qualitatively but lacked objective measurement scales. Adhesion formation was even less consistently reported, with only Bindal et al. noting the absence of adhesions during a one-year follow-up. This underreporting, likely due to the variability in follow-up durations and the subjective nature of pain assessments, limited the inclusion of these parameters in our primary outcomes.

One important limitation of our analysis was the inconsistency and heterogeneity in mesh reporting across studies. Therefore, we omitted mesh-based subgroup analyses to avoid misleading interpretations. This reflects a broader issue in robotic hernia surgery literature: despite technical advances, outcome reporting remains inconsistent. Establishing standardized mesh classification and reporting guidelines is crucial to improving scientific rigor and comparability.

Finally, none of the included studies reported on patient satisfaction, highlighting a critical gap in the current literature and the need for future studies to incorporate standardized patient-reported outcome measures.

## Conclusion

In conclusion, both r-IPOM and r-TAPP are safe and effective approaches for robotic ventral hernia repair, achieving high rates of defect closure. Our meta-regression identified a significant inverse association between closure rates and overall complication rates, underscoring the importance of complete, tension-free closure in optimizing outcomes. While perioperative results were largely comparable, r-TAPP may confer a potential advantage in reducing surgical site infections, possibly due to lower rates of seroma and hematoma formation, although the underlying mechanisms remain to be clarified. Ultimately, the selection of surgical approach and mesh type should be individualized based on patient-specific factors, anatomical context, and surgeon expertise.

## Supplementary Information

Below is the link to the electronic supplementary material.

Supplementary Material 1

Supplementary Material 2

Supplementary Material 3

## Data Availability

All data analyzed during this study are included in this published article and its supplementary information files.

## Abbreviations

rIPOM: robotic-assisted intraperitoneal onlay mesh repair
rIPOM +: robotic-assisted intraperitoneal onlay mesh repair + defect closure
rTAPP: robotic-assisted transabdominal preperitoneal repair
RCT: randomized clinical trials
SSI: surgical site infection
e-PTFE: expanded polytetrafluoroethylene (Synecore Mesh)
PVDF: Polyvinylidene fluoride (Dynamesh Mesh)
PGA: Polyglycolic acid (Dexon Mesh)
P4HB: Poly-4-hydroxybutyrate (Phasix™ Mesh)
