# Supplementary material for: “Comparative safety and efficacy of robotic TAPP and IPOM techniques in ventral hernia repair: a systematic review and meta-analysis of Short-term Outcomes”
Source: Hernia. 2025 Aug 19;29(1):255. doi: 10.1007/s10029-025-03454-0 (PMC12364986; doi:10.1007/s10029-025-03454-0)
Supplement: Supplementary file 1 — Supplementary Material 1 [file 10029_2025_3454_MOESM1_ESM.docx]

## Supplementary Figure 1. Leave−One−Out Sensitivity Analysis Summary

| **Study ID** | **Study Removed** | **New Proportion** | **95% CI** | **Change** | **I²** | **Studies** |
| --- | --- | --- | --- | --- | --- | --- |
| **Overall** | **Overall (All Studies)** | **0.9697** | **[0.9487, 0.9906]** | **Reference** | **73.9%** | **n=15** |
| **Study 1** | Kudsi C 2020 r−IPOM | 0.9763 | [0.9567, 0.9960] | +0.0066 | 69.1% | n=14 |
| **Study 2** | Kudsi R 2020 r−IPOM | 0.9708 | [0.9502, 0.9914] | +0.0012 | 74.2% | n=14 |
| **Study 3** | Gockal 2019 r−IPOM | 0.9773 | [0.9580, 0.9965] | +0.0076 | 67.4% | n=14 |
| **Study 4** | Kennedy 2018 r−IPOM | 0.9667 | [0.9443, 0.9891] | −0.0030 | 75.7% | n=14 |
| **Study 5** | Chelliah 2024 r−IPOM | 0.9640 | [0.9389, 0.9890] | −0.0057 | 75.6% | n=14 |
| **Study 6** | Ferraro 2023 r−IPOM | 0.9691 | [0.9478, 0.9904] | −0.0006 | 75.7% | n=14 |
| **Study 7** | Bindal 2024 r−TAPP | 0.9661 | [0.9434, 0.9889] | −0.0035 | 75.7% | n=14 |
| **Study 8** | Kudsi C 2020 r−TAPP | 0.9700 | [0.9476, 0.9924] | +0.0003 | 74.2% | n=14 |
| **Study 9** | Kudsi R 2020 r−TAPP | 0.9690 | [0.9476, 0.9903] | −0.0007 | 75.7% | n=14 |
| **Study 10** | Gockal 2019 r−TAPP | 0.9723 | [0.9508, 0.9939] | +0.0027 | 73.0% | n=14 |
| **Study 11** | Kennedy 2018 r−TAPP | 0.9659 | [0.9430, 0.9888] | −0.0038 | 75.7% | n=14 |
| **Study 12** | Chelliah 2024 r−TAPP | 0.9631 | [0.9368, 0.9893] | −0.0066 | 70.8% | n=14 |

## Supplementary Figure 2. Leave−One−Out Sensitivity Analysis Summary

| **Study ID** | **Study Removed** | **New Proportion** | **95% CI** | **Change** | **I²** | **Studies** |
| --- | --- | --- | --- | --- | --- | --- |
| **Overall** | **Overall (All Studies)** | **0.0574** | **[0.0224, 0.0924]** | **Reference** | **61.4%** | **n=8** |
| **Study 1** | Kudsi C 2020 r−IPOM | 0.0602 | [0.0178, 0.1025] | +0.0028 | 64.4% | n=7 |
| **Study 2** | Kudsi R 2020 r−IPOM | 0.0573 | [0.0210, 0.0935] | −0.0001 | 66.5% | n=7 |
| **Study 3** | Gockal 2019 r−IPOM | 0.0572 | [0.0166, 0.0979] | −0.0002 | 62.6% | n=7 |
| **Study 4** | Ferraro 2023 r−IPOM | 0.0561 | [0.0204, 0.0918] | −0.0013 | 65.5% | n=7 |
| **Study 5** | Baur 2021 r−IPOM | 0.0447 | [0.0133, 0.0761] | −0.0127 | 48.4% | n=7 |
| **Study 6** | Kudsi C 2020 r−TAPP | 0.0676 | [0.0427, 0.0925] | +0.0102 | 0.0% | n=7 |
| **Study 7** | Kudsi R 2020 r−TAPP | 0.0543 | [0.0202, 0.0884] | −0.0031 | 62.6% | n=7 |

# Supplementary Figure 3. Combined Complications Analysis by Mesh Type

**Summary: Events/Total (Proportion) by Mesh Type**

| **Mesh Type** | **Recurrence** | **Hematoma** | **Seroma** | **SSI** | **Reoperation** |
| --- | --- | --- | --- | --- | --- |
| Macroporous Mesh | 0/571 (0.000) | 19/673 (0.028) | 27/474 (0.057) | 10/585 (0.017) | 1/450 (0.002) |
| Mixed/Coated Mesh | 0/39 (0.000) | 0/7 (0.000) | 1/7 (0.143) | N/A | 0/39 (0.000) |

**Mesh Type Classification Reference**

| **Study** | **Original Mesh Description** | **Classification** |
| --- | --- | --- |
| Bindal 2024 | macroporous polypropylene | Macroporous Mesh |
| Kudsi R 2020 | N/A | Not Specified |
| Gockal 2019 | Macroporous 96.1%, Microporous 1.9% | Macroporous Mesh |
| Kennedy 2018 | Macroporous Polypropylene | Macroporous Mesh |
| Chelliah 2024 | monofilament, macroporous | Macroporous Mesh |
| Ferraro 2023 | Coated 27, Macroporous 6 | Mixed/Coated Mesh |
| Baur 2021 | Macroporous (DynameshEndolapVisible, Progrip) | Macroporous Mesh |
| Bauer 2024 | PVDF | PVDF Mesh |
| Kudsi C 2020 | Mainly Macroporous, some ePTFE | Macroporous Mesh |

# Meta−Regression Statistical Analysis

**Linear Regression Results**

**Supplementary Figure 4**

##### Unweighted Linear Regression:

Equation: Complication Rate = 0.4508 + −0.3715 × Closure Rate R² = 0.244, p−value = 0.1226

95% CI for slope: [−0.8646, 0.1217]

##### Sample Size−Weighted Regression:

Equation: Complication Rate = 1.0290 + −1.0121 × Closure Rate R² = 0.747, p−value = 0.0006

95% CI for slope: [−1.4565, −0.5676]

##### Correlation Analysis:

Pearson correlation: r = −0.494

95% CI: [−0.844, 0.151], p−value = 0.1226

##### Technique−Specific Results:

r−IPOM: r = −0.688, p = 0.1991 (n = 5 studies) r−TAPP: r = −0.415, p = 0.4131 (n = 6 studies)

##### Clinical Interpretation:

Higher closure rates are associated with LOWER complication rates

This suggests that achieving complete hernial defect closure may reduce complications
