# Supplementary material for: “Comparative safety and efficacy of robotic TAPP and IPOM techniques in ventral hernia repair: a systematic review and meta-analysis of Short-term Outcomes”
Source: Hernia. 2025 Aug 19;29(1):255. doi: 10.1007/s10029-025-03454-0 (PMC12364986; doi:10.1007/s10029-025-03454-0)
Supplement: Supplementary file 2 — Supplementary Material 2 [file 10029_2025_3454_MOESM2_ESM.docx]

| Population | Intervention | Outcome |
| --- | --- | --- |
| Hernia  Ventral hernia | Robotic  IPOM  IPOM+  Intraperitoneal onlay mesh  Intraperitoneal onlay mesh plus  TAPP  Transabdominal preperitoneal repair  Preperitoneal  r-IPOM | Operative time  Intraoperative bleeding  recurrence  morbidity  closure of hernial defect  Excision of hernial sac  Closure of hernial defect  Reoperation  Seroma  Hematoma    Surgical site infection  Console time  Length of hospital stay |

Search strategy (Medical subject headings (MeSH) and related terms)
