# Supplementary figures and images for: “Comparative safety and efficacy of robotic TAPP and IPOM techniques in ventral hernia repair: a systematic review and meta-analysis of Short-term Outcomes”
Source: Hernia. 2025 Aug 19;29(1):255. doi: 10.1007/s10029-025-03454-0 (PMC12364986; doi:10.1007/s10029-025-03454-0)

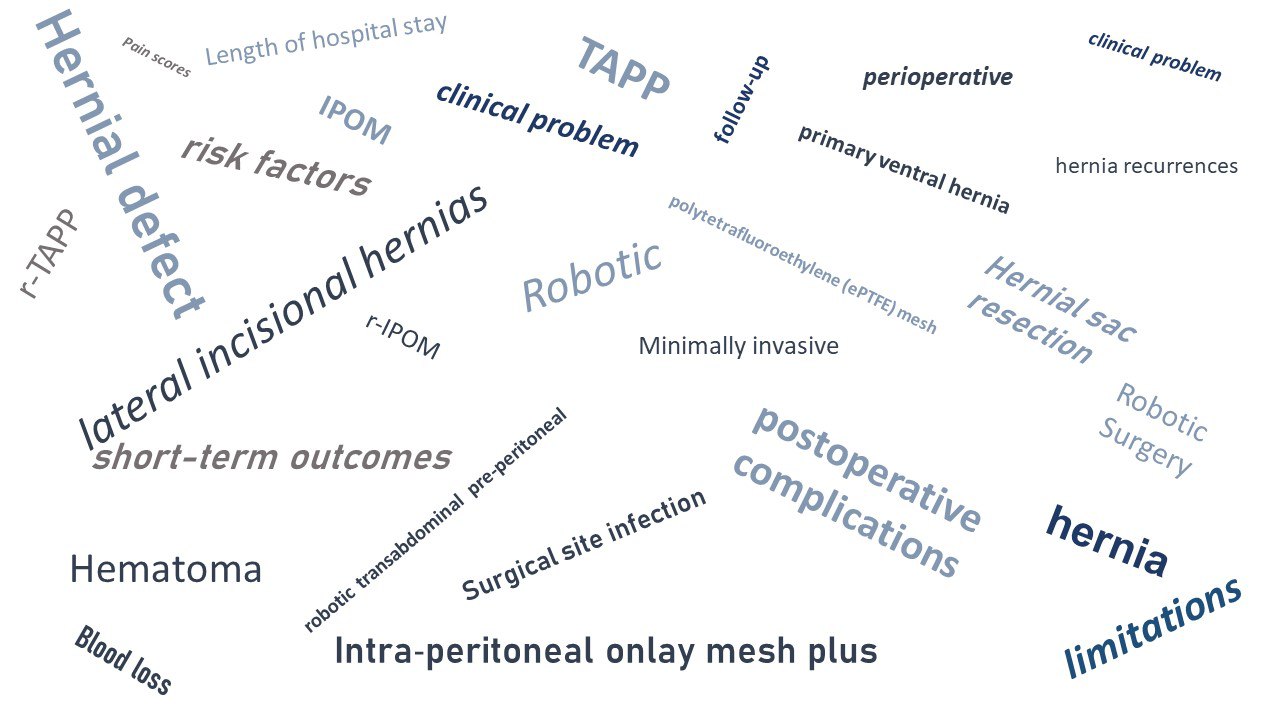

Supplement: Supplementary file 3 — Supplementary Material 3 [file 10029_2025_3454_MOESM3_ESM.jpg]
